# Chronic Release of Tailless Phage Particles from Lactococcus lactis

**DOI:** 10.1128/AEM.01483-21

**Published:** 2022-01-11

**Authors:** Yue Liu, Svetlana Alexeeva, Herwig Bachmann, Jesús Adrián Guerra Martínez, Nataliya Yeremenko, Tjakko Abee, Eddy J. Smid

**Affiliations:** a Food Microbiology, Wageningen University & Research, Wageningen, the Netherlands; b NIZO B.V., Ede, the Netherlands; c Amsterdam UMC, University of Amsterdam, Department of Rheumatology & Clinical Immunology and Department of Experimental Immunology, Amsterdam, the Netherlands; d TI Food and Nutrition, Wageningen, the Netherlands; University of Nebraska-Lincoln

**Keywords:** membranes, *Siphoviridae*, dairy starter culture, lipid bilayer, nonlytic phage release

## Abstract

Lactococcus lactis strains residing in the microbial community of a complex dairy starter culture named “Ur” are hosts to prophages belonging to the family *Siphoviridae*. L. lactis strains (TIFN1 to TIFN7) showed detectable spontaneous phage production and release (10^9^ to 10^10^ phage particles/ml) and up to 10-fold increases upon prophage induction, while in both cases we observed no obvious cell lysis typically described for the lytic life cycle of *Siphoviridae* phages. Intrigued by this phenomenon, we investigated the host-phage interaction using strain TIFN1 (harboring prophage proPhi1) as a representative. We confirmed that during the massive phage release, all bacterial cells remain viable. Further, by monitoring phage replication *in vivo*, using a green fluorescence protein reporter combined with flow cytometry, we demonstrated that the majority of the bacterial population (over 80%) is actively producing phage particles when induced with mitomycin C. The released tailless phage particles were found to be engulfed in lipid membranes, as evidenced by electron microscopy and lipid staining combined with chemical lipid analysis. Based on the collective observations, we propose a model of phage-host interaction in L. lactis TIFN1 where the phage particles are engulfed in membranes upon release, thereby leaving the producing host intact. Moreover, we discuss possible mechanisms of chronic, or nonlytic, release of LAB *Siphoviridae* phages and its impact on the bacterial host.

**IMPORTANCE** In complex microbial consortia such as fermentation starters, bacteriophages can alter the dynamics and diversity of microbial communities. Bacteriophages infecting Lactococcus lactis are mostly studied for their detrimental impact on industrial dairy fermentation processes. In this study, we describe a novel form of phage-bacterium interaction in an L. lactis strain isolated from a complex dairy starter culture: when the prophages harbored in the L. lactis genome are activated, the phage particles are engulfed in lipid membranes upon release, leaving the producing host intact. Findings from this study provide additional insights into the diverse manners of phage-bacterium interactions and coevolution, which are essential for understanding the population dynamics in complex microbial communities like fermentation starters.

## INTRODUCTION

Bacteriophages, viruses that infect bacteria, are highly diverse in shape, structure, and composition. They can be icosahedral, spherical, pleomorphic, filamentous, and droplet, bottle, and spindle shaped; some have a long or short tail, and some are tailless, engulfed in a lipid bilayer or containing lipids beneath the protein capsid; the genetic material can be double-stranded or single-stranded DNA or RNA (1, 2). The broad accessibility of high-throughput sequencing technologies also revealed a high degree of genetic diversity in bacteriophages; mosaic genomes and numerous novel sequences of unknown function have been reported (3–6). Over 90% of reported phages are tailed double-stranded DNA phages belonging to the order *Caudovirales* (7). Tailed phages primarily interact with their host cell by using tail fibers and baseplate structures and use the tail for penetrating the bacterial cell surface and viral DNA injection (8, 9). At the end of the infection cycle, virulent tailed phage particles are released from the cells by holin-lysin-induced lysis of the host. So-called temperate bacteriophages undergo an alternative, lysogenic cycle in which the bacteriophage DNA integrates into the chromosome of the host, becoming a prophage (10, 11). In this dormant state, the prophage can replicate its genome as a part of the bacterial chromosome. Under conditions insulting its host’s DNA integrity, the prophage can enter the lytic cycle, meaning that it excises from the bacterial chromosome, replicates its genome, assembles into mature phage particles, and escapes the host following phage holin-lysin-induced cell burst (12, 13).

About 4% of the described bacteriophages lack genes encoding tail proteins, and they represent polyhedral, filamentous, or pleomorphic phages (3, 7). Some members of this group also apply alternative strategies to release their progeny from infected bacteria. Filamentous phages of the *Inoviridae* family are assembled at the cell surface and excreted from infected cells continuously by extrusion, a process mediated by membrane translocation and channel proteins that leaves the host cells fully viable (14, 15). Another distinct mechanism of progeny release is budding, a delicate mechanism typical for animal viruses. During budding these viruses are encapsulated by the cell membrane and released without killing the host. So far, budding has been suggested only for the family *Plasmaviridae*, tailless phages infecting wall-less bacterial *Acholeplasma* species via membrane fusion (16, 17). In contrast to lytic phage release that kills the host, the nonlytic release is also referred to as chronic release (18). The group of tailless phages includes bacteriophages that have, in addition to nucleic acid and proteins, internal or external lipid constituents, a property originally associated with viruses infecting multicellular eukaryotes. Currently, the lipid-containing bacteriophages are classified into four families, *Corticoviridae*, *Cystoviridae*, *Plasmaviridae*, and *Tectiviridae* (19).

In complex microbial communities, for instance, the ones from the marine environment, the gastrointestinal tract, and in complex food fermentations, bacteriophages have impacts on the dynamics and diversity of microbial communities, and the bacterium-phage interactions play a key role in the evolution of both partners in the interaction (20, 21). Lactic acid bacteria (LAB) historically have been used in food fermentation, among which Lactococcus lactis plays important roles in various dairy fermentations. Phages infecting lactic acid bacteria (LAB), particularly L. lactis, are among the most studied for their detrimental impact on industrial (dairy) fermentation processes; phage activities may result in low/inconsistent quality of products and even failure of the whole fermentation (22). However, recent insights have revealed that the impacts of phages on the bacterial hosts are not all negative (20, 23).

Earlier, we described (pro)phages abundantly released and coexisting within a naturally evolved microbial community, a mixed (originally undefined) complex starter culture (named Ur) of LAB used in dairy fermentations (23). These cultures represent an interesting model ecosystem because it was established through long-term propagation by back-slopping. Practicing back-slopping creates the boundaries for natural selection, which drives adaptive evolution of the culture and its constituent microbial strains. Although (pro)phages are not desired in industrial fermentations with defined starter compositions, the common presence of prophages in the naturally evolved, stable, and robust starter cultures like Ur suggests an evolutionary success (6, 23). Investigating the behavior of phages and bacteria in such a model system contributes to the understanding of bacterium-phage interactions as well as its ecological and evolutional significance.

Based on analysis of the genomic content, the isolated (pro)phages from the Ur starter culture belong to P335 group lactococcal phages of the *Siphoviridae* family, order *Caudovirales*. In fact, all currently known phages infecting LAB are members of the *Caudovirales* order (7), or tailed phages. However, (pro)phages in this starter culture possess some peculiar features: phage particles are abundantly released spontaneously and further stimulated by mitomycin C induction (23). The phage particles appear to be tailless due to disruptions in tail-protein-encoding genes (6). Moreover, the release of the (pro)phages from the host cells was not accompanied by detectable cell lysis, a phenomenon that is typical for release of *Siphoviridae* bacteriophages (24–27). We set out to investigate this phenomenon in this study, and we demonstrate that the tailless *Siphoviridae* phage particles are enclosed in lipid membrane and are released from the cells by a nonlytic mechanism.

## RESULTS

### No detectable cell lysis during phage release.

A previous study on Lactococcus lactis strains TIFN1 to -7, originating from the mixed cheese starter culture, indicated no obvious drop in the optical density of the bacterial cultures following prophage activation (23). We were triggered by this observation and therefore further examined this phenomenon using strain TIFN1 as a model. We first analyzed the cell viability in prophage-induced cultures supplemented with mitomycin C (MitC) and control cultures without MitC induction. The total cell counts determined using a hemocytometer and the number of culturable cells, i.e., CFU, revealed nonsignificantly different, albeit slightly lower, average cell counts in the prophage-induced cultures (see Fig. S1 in the supplemental material), indicating that at least the vast majority of TIFN1 cells present under the tested conditions remained viable. Both cell counting methods showed no obvious differences in cell numbers from the prophage-induced cultures and noninduced control cultures, further confirming that in the L. lactis strains, represented by lysogen TIFN1, there was no detectable cell lysis in spite of the abundant phage release upon phage induction.

### The major part of the culture actively produces phages.

**FIG 1:**
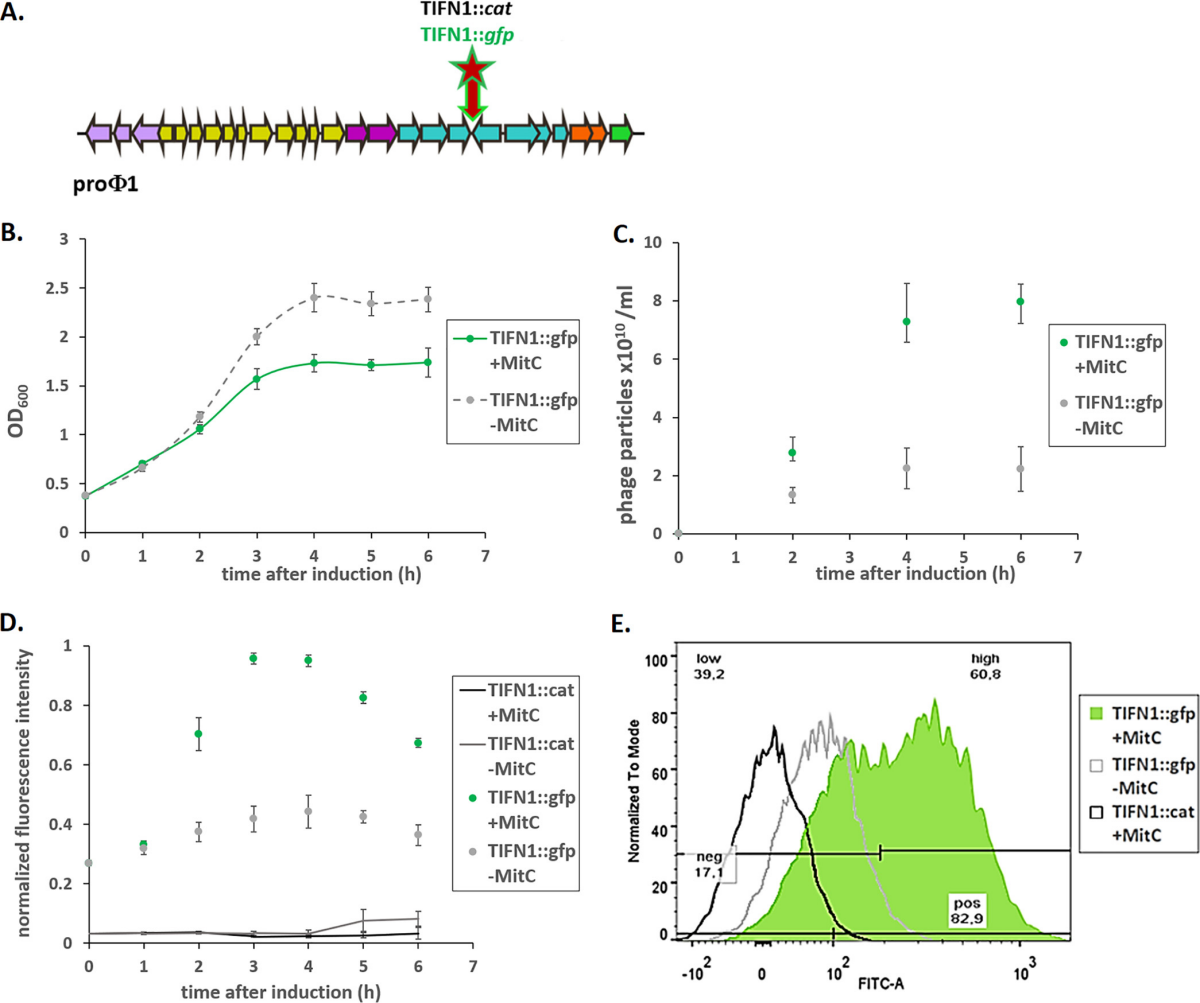
Phage labeling and examination of phage replication. (A) Schematic drawing of the prophage genome with marked *gfp* and *cat* insertion sites. Arrows represent ORFs and indicate the direction of gene transcription. The number of arrows does not reflect the real ORF numbers but is only a schematic presentation. The insertion was made between two convergent ORFs. Colors in arrows schematically represent different phage gene clusters. (B) Growth response of TIFN1::*gfp* to MitC treatment. (C) Phage release by TIFN1::*gfp* during MitC induction. Green symbols represent MitC-treated cultures, and gray symbols represent control cultures without MitC. (D) Dynamics of phage replication (as derived from average cell fluorescence intensity) during MitC induction (green symbols) and in uninduced samples (gray symbols) in reporter strain (TIFN1::*gfp*) compared to baseline fluorescence of non-*gfp* cultures (TIFN1::*cat*, black and gray lines for induced and uninduced conditions, respectively). (E) Fluorescence distribution in the population at 3 h of induction in non-*gfp* TIFN1::*cat* (black unfilled), uninduced TIFN1::*gfp* (gray unfilled), and MitC-induced TIFN1::*gfp* (green filled) cultures. The statistics in panel E are shown for induced TIFN1::*gfp*: 82.9% of the population was positive for green fluorescence (pos 82.9), 17.1% was fluorescence negative (neg 17.1), 60.8% was highly fluorescent (high 60.8), and 39.2% was low in fluorescence (low 39.2).

To elucidate whether phage production is a population-wide activity in a clonal culture of strain TIFN1, we monitored *in vivo* phage replication using a reporter strain, in which a *gfp* reporter was inserted within the prophage. In cells actively replicating the phage particles, green fluorescence intensity was expected to increase. As mentioned, we used L. lactis TIFN1 as the model strain, which harbors the genome of prophage proPhi1 (6). The insertion site was selected within the prophage sequence between stop codons of open reading frames (ORFs) 48 and 49 located on opposite DNA strands (Fig. 1A), resulting in the TIFN1::*gfp* strain. In parallel, a fluorescence negative-control strain was constructed in which the chloramphenicol resistance gene *cat* was inserted at the same site, yielding the TIFN1::*cat* strain.

The derived TIFN1::*gfp* strain showed growth behavior (Fig. 1B) similar to that of wild-type TIFN1 (see data in Alexeeva et al. [23]), where prophage induction by MitC led to a merely slight inhibition in growth instead of a decay in biomass, as indicated by monitoring culture turbidity. The TIFN1::*gfp* strain also produced phage particles (Fig. 1C) to an amount similar to that of the wild type (see data in Alexeeva et al. [23]): 10^10^ phage particles/ml were found in cultures without added MitC and phage numbers increased to ∼10^11^/ml upon MitC induction in 6 h, as estimated by quantifying phage DNA content.

To study the *in vivo* dynamics of prophage induction in L. lactis TIFN1, we used the TIFN1::*gfp* strain and monitored the fluorescence intensity of the cells by flow cytometry in MitC-induced and uninduced cultures. As a fluorescence negative control, we used the TIFN1::*cat* strain. The TIFN1::*cat* strain exhibited very low background fluorescence, not changing in time and not affected by MitC addition (Fig. 1D). The uninduced culture of the TIFN1::*gfp* strain showed moderate fluorescence (time point 0 h), and the fluorescence increased slightly in time. This is in line with the observed constitutive phage induction and replication taking place even without MitC induction (Fig. 1C and D). The induced culture of the TIFN1::*gfp* strain showed a clear increase in fluorescence intensity until the 4th hour postinduction, and then the fluorescence intensity declined slightly. The increase in fluorescence intensity of the cells was 2.5- to 3-fold and correlated with the increase in the number of released phage particles (Fig. 1C and D).

To examine whether the major fraction of the bacterial population actively produces phage particles, the distribution of fluorescence in individual cells was measured by flow cytometry. The fluorescence distribution per particle in the negative control (TIFN1::*cat*, black unfilled) as well as in uninduced cultures of TIFN1::*gfp* strain (gray unfilled) and MitC-induced TIFN1::*gfp* strain (green filled) at 3 h postinduction was measured (Fig. 1E). In the MitC-induced TIFN1::*gfp* culture, more than 80% of the cells are green fluorescent and more than 60% are highly fluorescent, which is a distinct population (second green peak in Fig. 1E). This indicates that the majority of the cells in the population actively replicate phage DNA and produce phage proteins. When relating this observation to the cell count results (Fig. S1), where the phage-inducing condition led to only slight growth inhibition rather than drastic decay in optical density, the hypothesis of nonlytic phage release is supported.

### Phages are enclosed in lipid bilayers.

**FIG 2:**
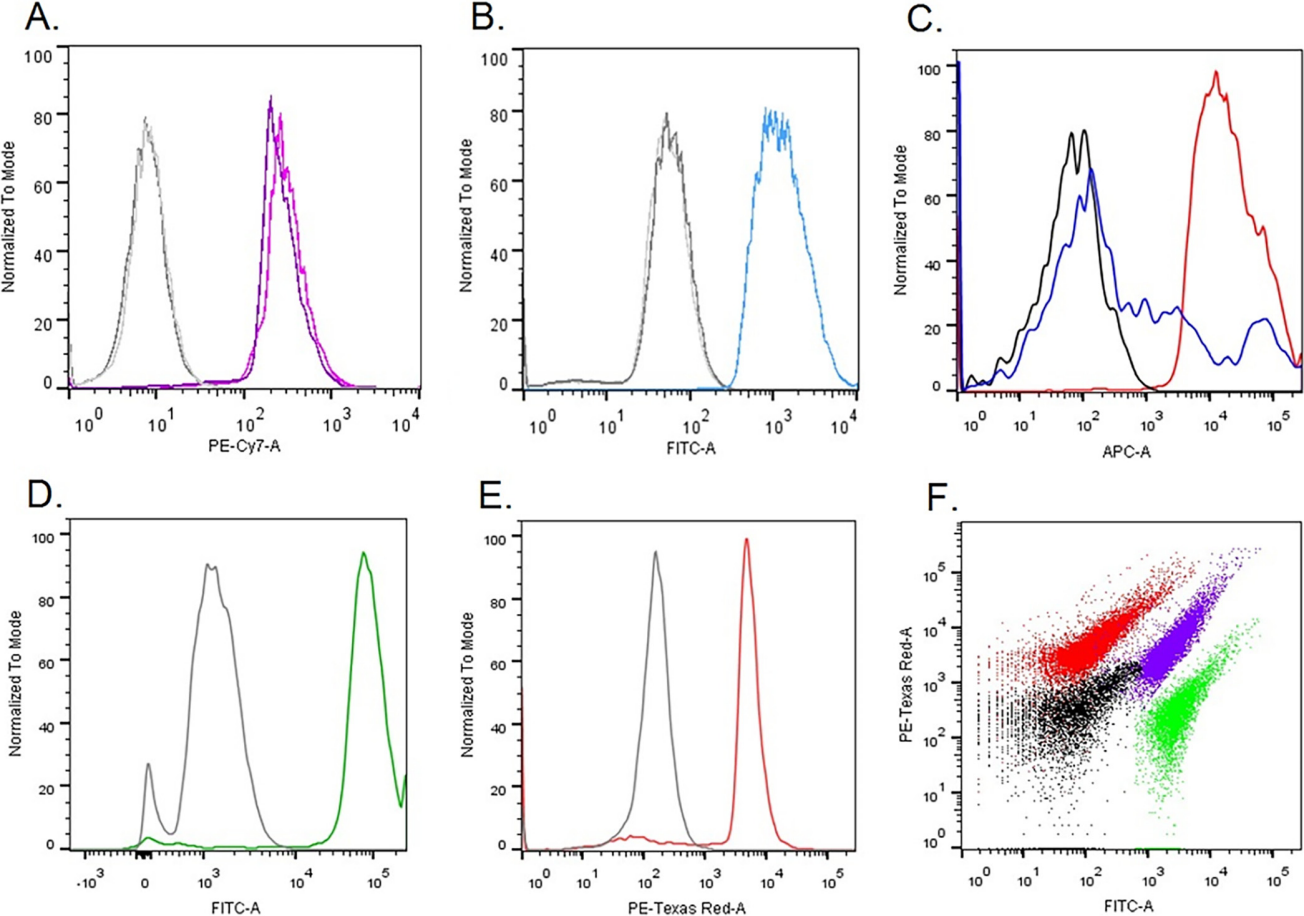
Staining proPhi1 particles with various lipophilic (A, B, C, and F) and DNA binding (D, E, and F) dyes followed by flow cytometry analysis. Gray/black, unstained phage particles. (A) Lipophilic dye 1. (B) Lipophilic dye 2. (C) Lipophilic dye 3. The blue line represents the sample stained after chloroform treatment. (D) DNA dye 1. (E) DNA dye 2. (F) Superimposed dot plot of proPhi1 particle samples with different staining: unstained (black), lipophilic dye 2 (green), DNA dye 2 (red), and double-stained lipophilic dye 2 and DNA dye 2 (purple-blue).

Nonlytic, chronic phage release has been previously described to occur via budding (*Plasmaviridae*) or extrusion (*Inoviridae*) (14–17). In case the budding mechanism of cell exit is recruited by the phage particles, it is expected to be enveloped by cellular lipids upon release. Therefore, first of all, we analyzed the presence of a lipid bilayer in/engulfing the phage particles. We employed three lipophilic dyes staining cellular membranes/lipid bilayers, but all were essentially nonfluorescent in aqueous media. All three lipophilic dyes were efficiently staining the phage particles (Fig. 2A to C), confirming the presence of lipid membranes. Moreover, when the phage particles were treated with chloroform prior to staining with lipophilic dye 3, the fluorescence was largely abolished (Fig. 2C, blue line). The chloroform-treated particles were visualized by electron microscopy (EM) and showed morphology typical of phage heads (see Fig. 1A and B from Alexeeva et al. [23]). We further confirmed that the lipid-enclosed particles are indeed bacteriophages containing DNA: the phage particles were readily stained with the two DNA dyes (Fig. 2D and E). Moreover, double staining with DNA dye 2 (red fluorescence) in combination with lipophilic dye 2 (membrane stain, green fluorescence) resulted in double-stained particles, confirming that the phage particles indeed contain DNA and are enclosed by membranes (Fig. 2F). This conclusion is also supported by the previous study where the tailless phage particles were isolated with the same method and subjected to DNA sequencing, and full phage genomes were recovered with more than 100-fold higher coverage than the background (6).

**FIG 3:**
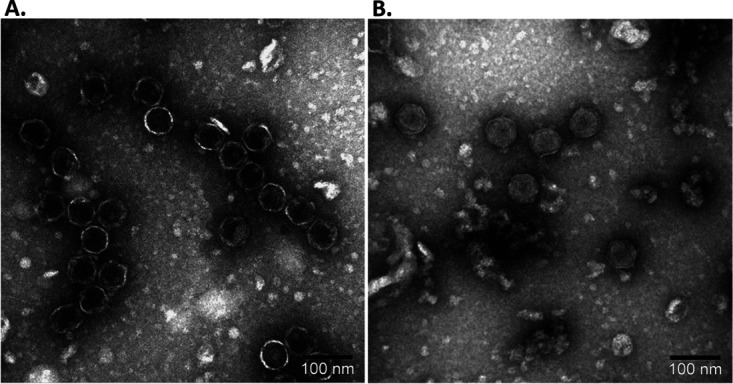
Transmission electron micrograph of proPhi1 with (A) and without (B) chloroform treatment.

Since the hypothesis of phage particles being enclosed in lipid bilayer is now supported by experimental evidence, we continued to find additional support by studying the phage particles with transmission electron microscopy (TEM). In this case, phage particles were not pretreated with chloroform to retain the lipid membrane, and we compared the particle morphology and size to chloroform-treated phage particles. It was observed that the morphology of untreated (Fig. 3A) and chloroform-treated (Fig. 3B) particles was similar, although they did show different electron densities, as reflected by the different darkness of particles, possibly indicating differences in compositions as chloroform will disintegrate lipid bilayers and dissolve lipids. We also noticed a difference in particle sizes caused by chloroform treatment. When measuring the particle diameters (defined as the distance between two opposite corners of the hexagon shape, measured by ObjectJ), untreated particles showed diameters of 65.4 ± 4.1 nm (*n* = 54), significantly (*P* < 0.00001, 2-tailed, unequal variance) larger than chloroform-treated particles with diameters of 58.0 ± 2.0 nm (*n* = 27). The difference in average diameters, 7.4 ± 4.6 nm, coincides with the thickness of two lipid bilayers (28). This analysis also supports the hypothesis that the released phage particles of L. lactis TIFN1 are enclosed in lipid bilayers.

### Lipid composition of phage particles differs from host cells.

**FIG 4:**
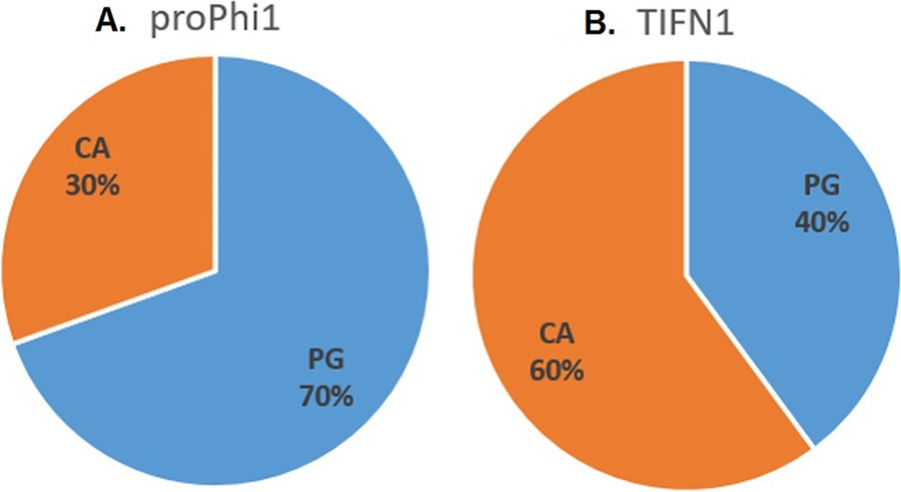
Phage and cell lipid composition. Composition of lipids extracted from isolated proPhi1 phage particles (A) and TIFN1 whole-cell-derived protoplast (B). PG, phosphatidyl glycerol; CA, cardiolipin.

Next, we extracted the lipids from phage crops produced by strain TIFN1 and also from whole-cell-derived protoplasts and subjected them to chemical lipid analysis using liquid chromatography coupled with mass spectrometry (LS-MS). As a phage-free control, phage-cured strain TI1c (23) was subjected to the same procedure of prophage induction and purification from the culture supernatant. The TIFN1 phage specimen lipid signals were well above the background level of the phage-free control from TI1c (Fig. S2). Phosphatidyl glycerol (PG) and cardiolipin (CA) were detected in phage samples as well as in cellular lipid samples; however, the ratio between the two major lipid species differed between the phage and the cell membrane lipid samples (Fig. 4).

The major lipid in the L. lactis cell membrane is cardiolipin, and a CA/PG ratio of about 2.2 has been determined for L. lactis membrane earlier (29). We found the CA/PG ratio value of 1.5 for cellular lipids extracted from TIFN1 (Fig. 4B). Remarkably, lipids of the phage crops were enriched in phosphatidyl glycerol with a CA/PG ratio of 0.4 (Fig. 4A). This suggests that the released phage particles are enclosed by phospholipids derived from distinct regions of lipid rafts/domains in the L. lactis cell membrane (30, 31).

**FIG 5:**
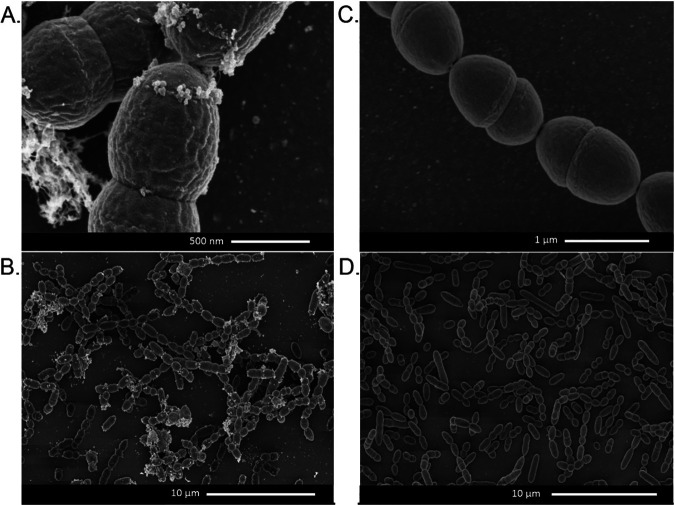
Scanning electron micrograph of cells subjected to 6-h MitC treatment. (A and B) TIFN1. (C and D) TI1c.

To further characterize the phage release from the cells we employed scanning electron microscopy to observe MitC-induced cells of wild-type strain TIFN1 and its prophage-cured derivative strain, TI1c (Fig. 5). The MitC treated TI1c had the usual morphology and smooth surface of a Gram-positive coccus without any detectable alteration (Fig. 5C and D). Strain TIFN1, however, showed a ruffled cell surface and accumulated numerous budlike, small spherical structures, typically near the cell division septum (Fig. 5A and B). The phage-cured strain TI1c lacked these extracellular structures.

From our observation that the lipid compositions differed between the released phages and the host cells, we speculated that the process of phage engulfing and release is specific for defined regions of the cell membrane; this speculation is further supported by the electron microscopic observation where the phage particles are accumulated near the cell division planes upon release.

## DISCUSSION

**FIG 6:**
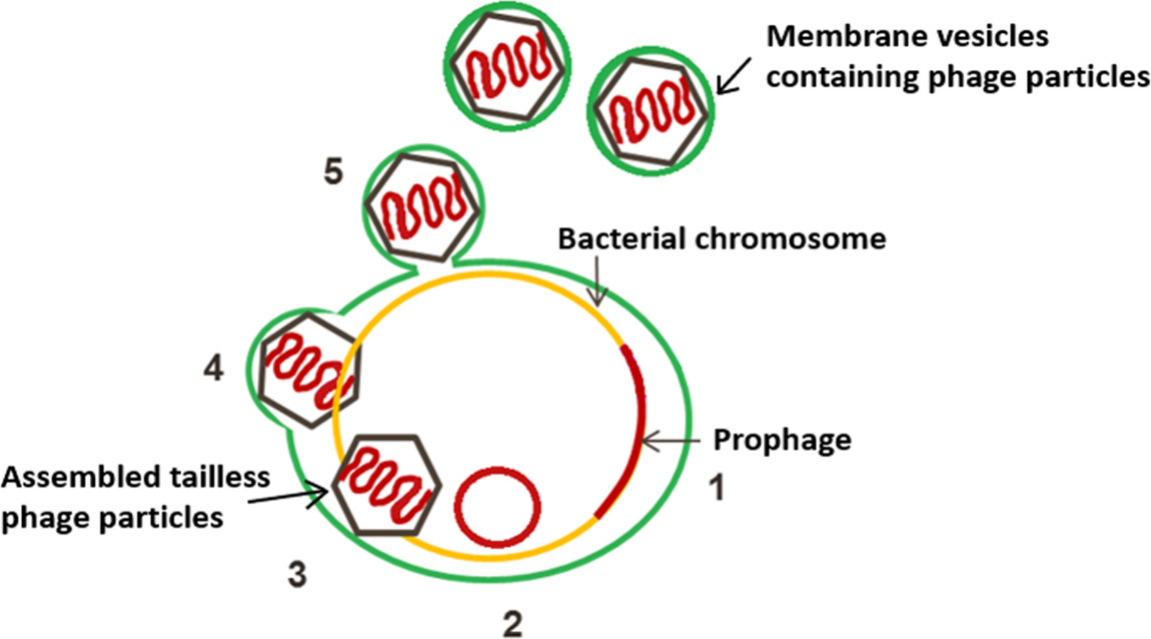
Schematic presentation of the proposed mechanism (steps 1 to 5) of phage release from Lactococcus lactis TIFN1. Activation of proPhi1 (steps 1 and 2) results in production of tailless *Siphoviridae* phage particles (step 3), enclosed in lipid membrane derived from the cytoplasmic membrane (green) (step 4), and released from the cells by a budding-like, nonlytic mechanism (step 5).

Bacteriophages are thought to be the most abundant biological entities on Earth and adopted a striking variety of forms and mechanisms of interaction with their host cells (32). Combining observations from this study, we propose a novel mechanism of interaction for lactococcal phages and their hosts, where the tailless *Siphoviridae* phage particles are enclosed in a lipid membrane and are released from the cells by a nonlytic mechanism (Fig. 6). This chronic, nonlytic phage release mechanism has not been previously described for LAB phages or *Siphoviridae* phages.

The prophage found in L. lactis TIFN1, referred to as proPhi1, is classified in the family of *Siphoviridae*, members of which are by definition tailed bacteriophages. Genomic analysis also revealed that genes encoding tail structures are present in these prophages, but, due to disruptions in some of the tail genes, the assembled phage particles show a tailless phenotype (6). Interestingly, the lipid-containing phages discovered so far, mostly assigned to families of *Corticoviridae*, *Cystoviridae*, *Plasmaviridae*, and *Tectiviridae*, are exclusively tailless phages (33). Plausibly, the reason that the membrane-containing feature was not found in any tailed phages is that they already achieve successful infection with the help of the tail device that efficiently penetrates the cell envelop, and no alternative infection mechanism was required (33). Tailless phages, on the other hand, have evolved to utilize the membrane to infect or interact with their hosts (33, 34). For example, enveloped phages use a membrane fusion mechanism to interact with the host and deliver their genetic materials (35–37). Therefore, it was part of the hypothesis that the tailless proPhi1 being enclosed in a lipid membrane could serve as an alternative infection strategy as the tail device is not available anymore, but we did not obtain evidence demonstrating the (re)infection of the host by the membrane-enclosed tailless phage particles (data not shown). It remains to be investigated whether the hurdle was for membrane-enclosed phage particles to attach and enter the host or, rather, for the tailless phage particles to inject their genetic material into the bacterial cytoplasm to complete the life cycle.

In previously described membrane-enclosed phages, the mechanism of incorporation of lipids to form virus-specific vesicles has been subjected to investigation, and hypotheses concerning underlying mechanisms have been proposed (33). For one, phage-encoded membrane proteins trigger cytoplasmic membrane formation in the host and enclose the phages during assembly in the cell. For example, *Cystoviridae* phage phi6 applies a mechanism in which the protein P9 was found to facilitate cytoplasmic membrane formation in bacteria (38). In this case, phage-encoded membrane proteins are incorporated into the host membrane, providing a scaffold for phage assembly, and the assembled phage particles are released upon lysis of the host (39, 40). Examples are *Tectiviridae* phage PRD1 employing membrane protein P10 (41) and *Corticoviridae* phage PM2 employing membrane proteins P3 and P6 to interact with phage-specific areas on the cell membrane (33, 42). For all above-mentioned phage-encoded proteins, we did not find homology to any of the proteins on proPhi1 or any of the other prophages found in lactococci isolated from the starter culture Ur (6). However, it should be noted that other membrane-associated protein-coding sequences were indeed predicted in the Ur prophages, namely, ORF42 in proPhi1 and proPhi5, ORF08 in proPhi2 and proPhi4, and ORF49 in proPhi6 (6). Targeting phage particles to special areas of cell membrane by membrane-associated proteins could be an explanation for the distinct lipid composition associated with released phage particles. Nevertheless, nonlytic release via a mechanism of budding still is not confirmed in other phages but suggested for plasmavirus (16) and is considered a very delicate life cycle of viruses, as it leaves the host alive while phages get to spread the progeny (33). Whether the nonlytic release of membrane-enclosed phages in L. lactis TIFN1 and other lactococcal strains found in the starter culture Ur is a result of long-term phage-host coevolution thus becomes an even more interesting hypothesis, especially as we observed similar growth behavior during phage release in other Ur strains (23).

Another intriguing question is how the membrane-enclosed phage particles escape from the bacterial host without lysis, especially given the fact that L. lactis is Gram positive, possessing a thick cell wall outside the cell membrane. A similar question has been raised for extracellular membrane vesicles (MVs or EVs) produced by Gram-positive bacteria ever since the discovery of such phenomena. It has already been known for a long time that *Archaea*, Gram-negative bacteria, and mammalian cells actively secrete the nanosized, lipid bilayer-enclosed particles, named EVs, harboring various nucleotide and protein cargos as a mechanism for cell-free intercellular interactions (43–45). Only recently, evidence was provided that EVs are also released by organisms with thick cell walls like Gram-positive bacteria, mycobacteria, and fungi (46–49), but the mechanistic insights are still lacking. Brown et al. (49) proposed several non-mutually exclusive mechanisms on the formation and release of EVs through thick cell walls, including the actions of turgor pressure, cell wall-modifying enzymes, and protein channels. The most evidence-supported mechanism is via cell wall-modifying enzymes, namely, autolysin (50) and prophage-encoded holin-endolysin (51, 52). Notably, phage particles have also been identified as part of the cargos in EVs produced by Bacillus subtilis (51). Further studies dedicated to elucidating the roles of autolysin and/or phage-encoded holin-endolysin in L. lactis TIFN1 would serve to reveal the release mechanism in this case.

Moreover, the effect of turgor pressure could also play a role in addition (49). It is plausible that upon prophage induction, the defective proPhi1 particles are abundantly assembled and accumulated in the cells, causing cytoplasmic crowding that results in elevated turgor pressure. The cell division site is often the target site of autolysins (53), in combination with induced phage-encoded endolysins, forming the weakest spot on the cell and giving opportunities for the phage particles to release under turgor pressure, which could explain our observation that the membrane-enclosed particles are mostly observed near the cell division sites and have a distinct lipid profile compared to the whole-cell samples. Therefore, we propose that the phenomenon of nonlytic membrane-engulfed phage release observed in L. lactis TIFN1 is driven by the concerted action of enzymatic activity and turgor pressure on the cell envelope, in combination with phage-encoded proteins to achieve phage-specific engulfment and release.

Although this is the first study to demonstrate nonlytic release of membrane-engulfed phages in LAB, we would like to point out that this could be a more common but overlooked phenomenon in other microbial communities for two reasons. First, studies focused on the detection of inducible prophages use cell lysis/plaque formation as a benchmark for phage activation. Obviously, when (tailless) phage particles are released via membrane envelops or other nonlytic ways, no apparent phenotype will be observed, thus discouraging further investigation. Second, it is a common practice in phage isolation protocol to employ chloroform to remove contaminating materials derived from bacterial cells (33); however, this treatment demolishes the membrane structures; therefore, the lipid-containing phenotype is conceivably not retrieved in further analysis of phage particles. We hope that our findings will inspire further studies, not only in elucidating the detailed mechanism of this case but also in awareness and discovery of similar phenomena in other microbial species and further shedding light on bacterium-phage interaction and coevolution.

### Conclusions.

In this study, we focused on a lysogenic L. lactis strain, TIFN1, isolated from a complex dairy starter culture as the model to examine phage-bacterium interactions. Employing a green fluorescent protein reporter, we monitored phage replication and release *in vivo* using flow cytometry. From this result, in combination with data on bacterial growth and phage particle quantification, we demonstrated that the majority of the bacterial population is actively producing phage particles when induced with mitomycin C while all bacterial cells remain viable. Evidence from electron microscopy, lipid staining, and chemical lipid analysis collectively suggests that the released tailless phage particles are engulfed in lipid membranes upon release, thereby leaving the bacterial host intact. Findings from this study provide additional insights into the diverse manners of phage-bacterium interactions, which is essential for understanding the population dynamics in complex microbial communities like fermentation starters and the coevolution of phages and bacteria.

## MATERIALS AND METHODS

### Strains and media.

Lactococcus lactis strains used in this study, including the wild-type strain TIFN1 isolated from a complex dairy starter culture named Ur (20), the prophage-cured derivative named TI1c (23), and the mutants created from strain TIFN1 in this study, namely, TIFN1::*cat* and TIFN1::*gfp* strains, were all statically cultivated in M17 broth (Oxoid) with 0.5% (wt/vol) lactose addition (Oxoid) at 30°C. All Escherichia coli strains harboring plasmids used in this study were cultivated in LB broth (BD Difco) supplemented with 150 μl/ml erythromycin and incubated at 37°C with aeration (shaking) at 150 × *g* in a shaker incubator.

### Cell growth, prophage induction, phage purification, and quantification.

Overnight cultures in M17 broth supplemented with 0.5% lactose (LM17) were diluted to an optical density at 600 nm (OD_600_) of 0.2 and allowed to grow for 1 h at 30°C before mitomycin C (MitC) was added (final concentration of 1 μg/ml). For control purposes, the same diluted cultures without MitC were used. Incubation proceeded for 6 h unless specified otherwise, and the turbidity (OD_600_) was monitored at 1-h intervals. At the end of induction, the total cell number was determined by direct counting in a hemocytometer chamber, and the viable count was made by standard spread plating on M17 agar supplemented with 0.5% lactose. Released phage particles were concentrated from the culture supernatants by polyethylene glycol (PEG)-NaCl precipitation as previously described (6), and the quantity was estimated based on phage DNA content in culture supernatants or in PEG-NaCl concentrated phage suspensions using agarose gel electrophoresis as previously described (23).

### Construction of plasmids for chromosomal integration into prophage sites.

Plasmid pSA114 is a derivative of plasmid pCS1966 (54), the chromosomal integration vector, allowing positive selection of cells in which the plasmid had been excised from the genome, resulting in unmarked integrations in the chromosome of L. lactis.

Two DNA fragments, 671 and 941 bp, of adjacent loci of prophage (proPhi1) were amplified from L. lactis TIFN1 chromosome using 1M_HR1_Fw+/1M_HR1_Rv and 1M_HR2_Fw/1M_HR2_1Rv+ primer pairs, respectively (Table 1). The two fragments were interconnected by a multiple cloning site (MCS) introduced by PCR overlap extension mutagenesis to allow further insertions between the homology arms. The resulting 1.7-kb PCR fragment was digested with KpnI and NcoI and ligated into corresponding sites of pSEUDO-GFP (55), resulting in plasmid pSA114. The 34-bp MCS between the amplified prophage sequences of pSA114 was used for further cloning. pSA116, the vector for integration of CmR (chloramphenicol resistance cassette, *cat*) into prophage, was made by inserting CmR between the prophage homology regions of pSA114. The chloramphenicol cassette (*cat*) was amplified by PCR using pGhCAM2_Fw/pGhCAM_Rv primers (Table 1) and pVE6007 (pGhost7 [56]) as a template. The fragment was digested with EcoRI and BamHI and subcloned into corresponding sites of pSA114, yielding pSA116. Plasmid pSA120, the vector for integration of *gfp* (the gene of the superfolder variant of GFP [57]) into prophage, was made as follows. The *gfp* flanked by CP25 artificial promoter (58) and two terminator sequences was excised from pIL-JK2 using EcoRI and BamHI and inserted into the same site (between the prophage homology regions) of pSA114, yielding pSA120.

**TABLE 1:** Primers used in this study^*a*^

Name	RE	Nucleotide sequence (5′–3′)	Product (bp)
1M_HR1_Fw+	MCS	GAATTCCCGGGTCGACAAGCTTAGATCTGGATCCTTGTTGGTTTTGGGCCCATCACTTTA	671
1M_HR1_Rv	NcoI	TTCCATGGGCGCTCCTTCAGGAAAGACGATTA	
1M_HR2_Fw	KpnI	TTGGTACCGGCGCTTGGTTATTCTGCTTCTGA	941
1M_HR2_1Rv+	MCS	GGATCCAGATCTAAGCTTGTCGACCCGGGAATTCTTTGGGTGGCCCATTTCCTACA	
pGhCAM2_fw	EcoRI	AAGAATTCAAGGGGATTTTATGCGTGAGAATG	984
pGhCAM_rv	BglII/XhoI/BamHI	ATGGATCCTCGAGATCTGAAAACCCTGGCGTTACCC	

a Restriction enzyme (RE) sites are underlined.

### Modification of the chromosomal integration strategy for industrial strains and construction of new integration vectors.

The designed vectors are derivatives of pCS1966 and unable to replicate in L. lactis. The original strategy (54) includes both transformation and chromosomal integration by homologous recombination for the successful acquisition of such vectors by L. lactis cells. Therefore, the plasmid acquisition is drastically dependent on the efficiency of transformation. Industrial wild strains usually feature poor transformability compared to domesticated laboratory L. lactis strains. Therefore, a new strategy has been designed by splitting plasmid transformation and its homologous recombination events. We constructed vectors that combine L. lactis thermosensitive (Ts) replication origin *repA^Ts^* and *oroP* genes. Ts replication origin allows plasmid replication under permissive temperature after the transformation event, followed by integration through homologous recombination at elevated temperature. Gene *oroP* enables counterselection for loss of the plasmid backbone, leaving unmarked integrations in the chromosome of L. lactis at specific target sites.

New vectors, pSA130-YL and pSA132-YL, were constructed as follows. E. coli strain EC1000 (59) was used for cloning and plasmid propagation. pG^+^host9 (56) was used to provide the backbone with *repA^Ts^* and *ermAM*. The plasmids pSA116 and pSA120 were used to provide the cassettes of DNA labels (CmR or *sf-gfp*) flanked by proPhi1 homology regions (MHR) and *oroP*. The KpnI/FspI fragment of pSA116 was ligated into KpnI/EcoRV-digested pG^+^host9, resulting in pSA130-YL. pSA132-YL was constructed in two steps: first, the KpnI/SalI fragment of pSA120 was ligated into the corresponding site of pG^+^host9, and then the SalI/FspI fragment of pSA120 was ligated into the SalI/EcoRV site, yielding pSA132-YL.

### Plasmid integration and backbone elimination.

L. lactis transformation was performed using a modified protocol as we described earlier (23). The confirmed transformants were propagated at the permissive temperature, 28°C, in M17 broth (0.5% glucose or lactose) with 3 μg/ml erythromycin and stored in 15% glycerol at −80°C until further use. For the integration step, L. lactis cells transformed with constructed plasmids were incubated at 37°C overnight, the OD_600_ of cultures was measured, cells were plated in proper dilutions (depending on OD_600_ values) on selection plates (L/GM17, 1.5% agar, 0.5 M sucrose and 3 μg/ml erythromycin), and cells were incubated at 37°C until colonies emerged. The presence and orientation of the whole-plasmid inserts were checked with PCR, and correct validated clones were maintained as 15% glycerol stocks at −80°C.

For the backbone elimination step, the validated clones with integrated plasmids were inoculated in 2 ml SA medium (60) containing 1% lactose or glucose and incubated at 30°C overnight. They then were diluted 10× in SA (1% lactose or glucose) medium and incubated at 30°C for 6 h. Thereafter, 10 μl of the culture was streaked on an SA (1% lactose or glucose) agar plate supplemented with 10 μg/ml 5-fluoroorotate (Sigma). Plates were incubated at 30°C until 5-fluoroorotate-resistant colonies emerged. For resulting labeled strains, TIFN1::*cat* and TIFN1::*gfp* strains (inserted genes from corresponding plasmid donors, pSA130-YL and pSA132-YL), correct inserts and their location on the TIFN1 chromosome were confirmed by PCR, sequencing the PCR products, and phenotypic analysis of either green fluorescence or chloramphenicol resistance.

### Phage lipid and DNA labeling.

The dyes used for labeling of lipids and DNA are shown in Table 2. To stain DNA, PEG-precipitated phage particles were incubated with Sybr green (no. S7563; Invitrogen, Molecular Probes) at 80°C for 10 min in a 10^−4^ final dilution of commercial stock as described earlier (61) or with GelRed nucleic acid gel stain (10^−4^ final dilution of commercial stock; Biotium) under the same conditions.

**TABLE 2:** Corresponding labeling of lipid and DNA dyes

Label used in text	Name
Lipophilic dye 1	FM 4-64
Lipophilic dye 2	MitoTracker green FM
Lipophilic dye 3	CellMask DeepRed
DNA dye 1	Sybr green
DNA dye 2	GelRed nucleic acid gel stain

For membrane detection, CellMask DeepRed (Life Technologies GmbH) was used in a final dilution of 10^−3^ of commercial stock, and phages were incubated for 5 min at 37°C. FM4-64 (Molecular Probes) was used at a final concentration of 20 μg/ml, and incubation proceeded for 15 min at room temperature. MitoTracker green FM (no. M7514; Molecular Probes) was used at a final concentration of 20 nM, and phages were incubated with the dye for 15 min at 37°C. For double staining of DNA and membrane, MitoTracker was added to the phages stained with GelRed, and the samples were vortexed and measured immediately by flow cytometry. In control experiments the membranes were first extracted by adding to the phage suspension equal volumes of chloroform. The samples were vortexed, centrifuged for 3 min at 14,000 × *g*, and aqueous phase containing the phage particles was collected. The staining was performed as described above.

When phages were not added, no detectable fluorescent particles were present in the control samples. To exclude contamination of phage suspension by bacterial cells, bacteria were added to phage suspension prior to staining of either DNA or lipids. In these control samples an additional population of particles with much higher fluorescence intensity was detected (not shown), as anticipated, given a bacterial cell contains much larger amounts of lipids and DNA per particle compared to phages.

### Flow cytometry.

Prior to flow cytometry analysis, 2 μl of fluorescent microspheres (1 × 10^−3^ of the stock Fluoresbrite YG microspheres; 0.75 μm; Polysciences) was added and the volume was adjusted to 500 μl by adding FACSFlow solution (10 mM phosphate-buffered saline, 150 mM NaCl, pH 7.4; Becton, Dickinson).

Samples were analyzed by using a BD FACSAria III flow cytometer (BD Biosciences, San Jose, CA). The cytometer was set up using an 85-μm nozzle and was calibrated daily using BD FACSDiva Cytometer Setup and Tracking (CS&T) software and CS&T beads (BD Biosciences). A 488-nm air-cooled argon-ion laser and the photomultipliers with 488/10-nm band pass filter for forward and side scatter (FSC and SSC) and with a 530/30-nm filter (with a 502 LP filter) was used for the detection of GFP, Sybr green, and MitoTracker. GelRed was excited with a yellow-green 561-nm laser and detected using a 610/20-nm filter with an LP 600-nm filter. CellMask DeepRed was excited with a 633-nm laser and detected with 660/20-nm filters. FM4-64 dye was excited with a 561-nm laser and detected at 780/60 nm with an LP 735-nm filter. FSC and SSC voltages of 300 and 350, respectively, and a threshold of 1,200 on FSC were applied to gate the bacteriophages and bacterial cell population.

### Chemical lipid analysis.

Normal-phase high-performance liquid chromatography (NP-HPLC) with evaporative light scattering detection (ELSD) was used for the quantitative analysis of phospholipids (62).

The analyses were performed with a 600E system controller (Waters), vacuum degasser (Knauer), 231 XL sampling injector (Gilson), and 3300 ELSD (Alltech). Extraction of the phospholipids from a freeze-dried sample was done with a mixture of chloroform, methanol, and ammonia (NH_3_) in water. After centrifugation of the sample for 10 min at 4,500 × *g*, 10.0 ml of the supernatant was evaporated under vacuum at 40°C in a heating block. When the sample was dried, 1 ml absolute ethanol was added and again evaporated to dryness. The dried sample was dissolved in 1.0 ml of the phospholipid solvent containing iso-octane, chloroform, and methanol at a ratio of 20:55:25, respectively.

Fifty milliliters of the sample solution was injected on an Xbridge amide analytical column, 3.5 μm, 4.6 by 250 mm (Waters). The components were eluted at a flow rate of 1.0 ml/min with a gradient of eluent A (iso-octane and acetone) and eluent B (2-propanol and ethyl acetate) to eluent C (2-propanol, water, ammonia and acetic acid) in 50 min.

1,2-Dipalmitoyl-sn-glycero-3-phosphoryl ethanolamine (PE; Matreya), 1,2-dipalmitoyl-sn-glycero-3-phosphoryl glycerol (PG; Matreya), 3-sn-lyso phosphatidyl ethanolamine (LPE; Sigma), dl-α-phosphatidyl choline (dipalmitoyl; C_16:0_) (PC, Sigma), sphingomyelin (SM; Sigma), phosphatidyl serine (oleoyl) (PS; Matreya), lysophosphatidylcholine (palmitoyl) (LPC; Matreya), and phosphatidylinositol (linoleoyl) (PI; Matreya) were used as calibration standards for quantitative analysis. A reference sample (buttermilk powder) with known amounts of phospholipids was analyzed, and recovery of spiked phospholipids was performed to control for accuracy and precision of the method.

### Scanning/transmission electron microscopy.

For scanning electron microscopy, L. lactis TIFN1 and TI1c cultures were subjected to 1 μg/ml MitC induction as described above. After 5 h of induction, the samples were fixed with 2.5% glutaraldehyde in phosphate-buffered saline (PBS) buffer for 1 h at room temperature. A droplet of the fixed cell suspension was placed onto poly-l-lysine-coated coverslips (Corning BioCoat, USA) and allowed to stand for 1 h at room temperature. After rinsing in PBS, the samples were poststained in 1% osmium tetroxide in PBS. Subsequently the samples were dehydrated in a graded series of ethanol, followed by critical point drying with CO_2_ (Leica EM CPD300; Leica Microsystems). The coverslips were fitted onto sample stubs using carbon adhesive tabs and sputter coated with 6-nm iridium (Leica SCD500). Samples were imaged at 2 kV, 6 pA, at room temperature in a field emission scanning electron microscope (Magellan 400; FEI Company, OR, USA).

For transmission electron microscopy, purified phage particles were subjected to negative staining and examined exactly as described previously (23).

### Data analysis.

The flow cytometry data were acquired by using BD FACSDiva software and analyzed by using FlowJo flow cytometry analysis software (Tree Star, Ashland, OR).

Lipid analysis (HPLC) data were analyzed with Chromeleon software, version 7.2 (Thermo Fisher Scientific). The nonlinear response of the ELSD was converted to a more linear signal to increase the accuracy of the quantification of phospholipids differing in fatty acid composition compared to those of the standard.

### Data availability.

The data sets used and/or analyzed during the current study are available from the corresponding author on reasonable request.
