# Supplementary material for: Chronic Release of Tailless Phage Particles from Lactococcus lactis
Source: Appl Environ Microbiol. 2022 Jan 11;88(1):e01483-21. doi: 10.1128/AEM.01483-21 (PMC8752148; doi:10.1128/AEM.01483-21)
Supplement: Supplemental file 1 — Fig. S1 and S2. Download AEM.01483-21-s0001.pdf, PDF file, 0.1 MB [file aem.01483-21-s0001.pdf]

Supplementary materials

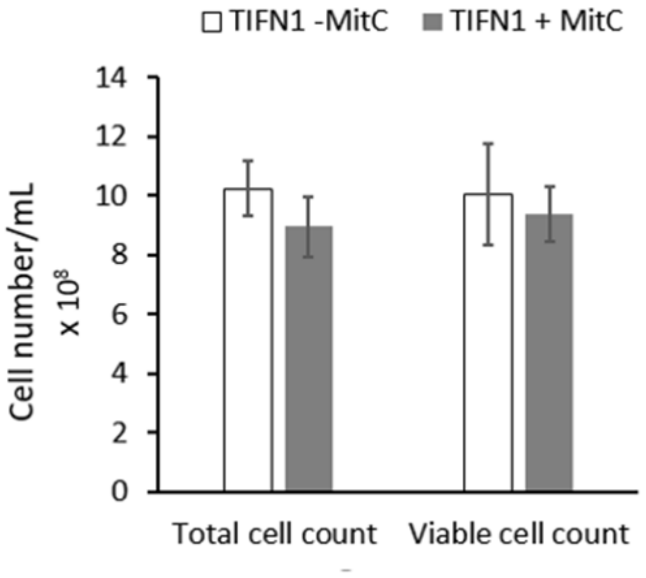

Figure S1. Cell count of *Lactococcus lactis* strain TIFN1 under phage induction conditions. Total cell count (obtained by counting cells using a haemocytometer) and viable cell count (determined by plating and colony count) in TIFN1 cultures induced with MitC at 7 hours and control cultures without induction.

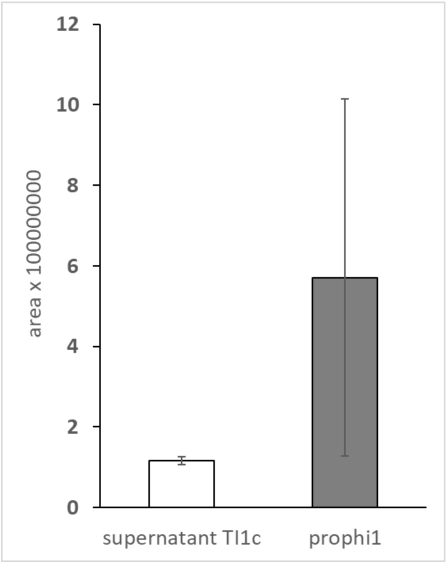

Figure S2. Lipid (sum of phosphatidyl glycerol and cardiolipin) signal detected in culture supernatant of phage-free control TI1c and proΦ1 collected from culture supernatant of TIFN1.
